# Justice and responsibility in climate change adaptation research

**DOI:** 10.2471/BLT.25.294163

**Published:** 2026-02-03

**Authors:** Kyle Ferguson, Caesar Alimsinya Atuire, Sonali Shukla McDermid, Rajesh Vedanthan

**Affiliations:** a Department of Philosophy, Hunter College, City University of New York, 695 Park Avenue, New York, NY 10065, United States of America (USA).; b Centre for Tropical Medicine and Global Health, University of Oxford, Oxford, England.; c Department of Environmental Studies, New York University, New York, USA.; d Institute for Excellence in Health Equity, NYU Grossman School of Medicine, New York, USA.

## Abstract

We address an ethical challenge in climate change adaptation and global health research. The challenge stems from two pairs of intuitions about justice and responsibility in climate change and health. One pair assigns responsibility for adaptation research to high-income countries given their historical emissions, disproportionate share of resources and capacity to intervene. The other pair assigns responsibility to low- and middle-income countries given their agency, right to self-determination, local authority and legitimacy, and disproportionate burden of climate and health risks. The intuitions create conflicting views: obligation and assistance pull in one direction, and agency and authority pull in another. To resolve the tension, we distinguish two forms of responsibility: (i) adaptation-enabling responsibilities; and (ii) adaptation-enacting responsibilities. The resulting division of labour reflects different forms of justice and aligns with the principle of subsidiarity’s core elements, namely: non-abandonment, non-absorption, and cooperation and coordination. We thus propose a framework that ascribes adaptation-enabling responsibilities to high-income countries, including adaptation financing, capacity-building and other forms of support; and adaptation-enacting responsibilities to low- and middle-income countries, including priority-setting in local adaptation research, and creation and implementation of their adaptation plans and policies. Our framework also suggests a third form of responsibility: shared adaptation responsibilities, which are jointly assigned to high-income countries, low- and middle-income countries and agents at multiple levels within them. We conclude that genuine collaboration in adaptation research, where high-income countries enable without dominating and low- and middle-income countries act without being abandoned, will be essential for just and effective adaptation to climate change.

## Résumé

Nous abordons un défi éthique dans le domaine de la recherche sur l’adaptation au changement climatique et la santé mondiale. Ce défi découle de deux intuitions concernant la justice et la responsabilité en matière de changement climatique et de santé. La première attribue aux pays à revenu élevé la responsabilité de la recherche sur l’adaptation, compte tenu de leurs émissions historiques, de la part disproportionnée de leurs ressources et de leur capacité d’intervention. La seconde attribue cette responsabilité aux pays à revenu faible et intermédiaire, compte tenu de leur capacité d’action, de leur droit à l’autodétermination, de leur compétence et légitimité locales, ainsi que du fardeau disproportionné que représentent les risques climatiques et sanitaires. Ces intuitions donnent lieu à des points de vue contradictoires: l’obligation et l’assistance vont dans une direction, tandis que la capacité d’action et les compétences vont dans l’autre direction. Pour remédier à cette tension, deux formes de responsabilité émergent: (i) les responsabilités qui permettent l’adaptation et (ii) les responsabilités qui la mettent en œuvre. La répartition des tâches qui en résulte reflète différentes formes de justice et correspond aux éléments fondamentaux du principe de subsidiarité, à savoir le non-abandon, la non-absorption, la coopération et la coordination. Nous proposons donc un cadre qui impute d’une part aux pays à revenu élevé les responsabilités liées à la facilitation de l’adaptation, notamment son financement, le renforcement des capacités et d’autres formes de soutien, et d’autre part aux pays à revenu faible et intermédiaire les responsabilités liées à la mise en œuvre de l’adaptation, notamment la définition des priorités dans la recherche locale sur l’adaptation, ainsi que l’élaboration et la mise en œuvre de leurs plans et politiques d’adaptation. Notre cadre suggère également une troisième forme de responsabilité: les responsabilités d'adaptation partagées, qui sont attribuées conjointement aux pays à revenu élevé, aux pays à revenu faible et intermédiaire et aux agents à plusieurs niveaux au sein de ceux-ci. En conclusion, il faudra une véritable collaboration dans la recherche sur l’adaptation, où les pays à revenu élevé procèdent à une facilitation sans dominer et où les pays à revenu faible et intermédiaire agissent sans être abandonnés, en vue d’une adaptation juste et efficace au changement climatique.

## Resumen

Se aborda un desafío ético en la investigación sobre la adaptación al cambio climático y la salud mundial. El desafío se deriva de dos pares de intuiciones sobre justicia y responsabilidad en el cambio climático y la salud. Un par asigna la responsabilidad de la investigación sobre la adaptación a los países de ingresos altos, dadas sus emisiones históricas, su cuota desproporcionada de recursos y su capacidad de intervención. El otro par asigna la responsabilidad a los países de ingresos bajos y medios, dada su capacidad de acción, su derecho a la libre determinación, su autoridad y legitimidad locales, y su carga desproporcionada de riesgos climáticos y sanitarios. Estas intuiciones generan visiones contrapuestas: la obligación y la asistencia apuntan en una dirección, y la capacidad de acción y la autoridad apuntan en otra. Para resolver esta tensión se distinguen dos formas de responsabilidad: (i) responsabilidades que posibilitan la adaptación; y (ii) responsabilidades que ejecutan la adaptación. La división del trabajo resultante refleja distintas formas de justicia y se alinea con los elementos centrales del principio de subsidiariedad, a saber: no abandono, no absorción, y cooperación y coordinación. Se propone así un marco que atribuye a los países de ingresos altos las responsabilidades que posibilitan la adaptación, incluida la financiación de la adaptación, el fortalecimiento de capacidades y otras formas de apoyo, y a los países de ingresos bajos y medios las responsabilidades que ejecutan la adaptación, incluida la definición de prioridades en la investigación local sobre la adaptación, así como la elaboración y aplicación de sus planes y políticas de adaptación. El marco también plantea una tercera forma de responsabilidad: las responsabilidades compartidas de adaptación, que se asignan conjuntamente a los países de ingresos altos, a los países de ingresos bajos y medios y a los agentes de múltiples niveles dentro de ellos. Se concluye que una colaboración genuina en la investigación sobre la adaptación, en la que los países de ingresos altos posibiliten sin dominar y los países de ingresos bajos y medios actúen sin ser abandonados, será esencial para una adaptación al cambio climático justa y eficaz.

## ملخص

نتناول هنا تحديًا أخلاقيًا في أبحاث التكيف مع تغير المناخ والصحة العالمية. ينبع هذا التحدي من وجود زوجين من الأفكار بشأن العدالة والمسؤولية في تغير المناخ والصحة. يضع أحدهما على عاتق الدول ذات الدخل المرتفع مسؤولية أبحاث التكيف نظرًا لأصولها التاريخية، وحصتها غير المتناسبة من الموارد، وقدرتها على التدخل. بينما يضع الآخر المسؤولية على عاتق الدول ذات الدخل المنخفض والمتوسط نظرًا لفاعليتها، وحقها في تقرير المصير، وسلطتها المحلية وشرعيتها، والعبء غير المتناسب الذي تتحمله من مخاطر المناخ والصحة. يُؤدي هذان الزوجين من الأفكار إلى تضارب في وجهات النظر: فالالتزام والمساعدة يجذبان في اتجاه، بينما تجذب القدرة على اتخاذ القرارات والسلطة في اتجاه آخر. ولحل هذا التضارب، فإننا نميز بين نوعين من المسؤولية: (أ) مسؤوليات تمكين التكيف؛ و(ب) مسؤوليات تنفيذ التكيف. يعكس هذا التقسيم الناتج للعمل أشكالًا مختلفة من العدالة، ويتماشى مع العناصر الأساسية لمبدأ التبعية، وهي: عدم التخلي، وعدم الاستيعاب، والتعاون والتنسيق. لذا، فإننا بذلك نقترح إطارًا للعمل يُسند مسؤوليات تمكين التكيف إلى الدول ذات الدخل المرتفع، بما في ذلك تمويل التكيف وبناء القدرات وأشكال الدعم الأخرى، ومسؤوليات تنفيذ التكيف إلى الدول ذات الدخل المنخفض والمتوسط، بما في ذلك تحديد الأولويات في أبحاث التكيف المحلية، ووضع خطط وسياسات التكيف الخاصة بها وتنفيذها. كما يقترح إطار العمل لدينا شكلًا ثالثًا من المسؤولية: مسؤوليات التكيف المشترك، الذي يُسند بشكل مشترك إلى الدول ذات الدخل المرتفع والدول ذات الدخل المنخفض والمتوسط والجهات الفاعلة على مستويات متعددة داخلها. ونخلص إلى أن التعاون الحقيقي في أبحاث التكيف، حيث تُمكّن الدول ذات الدخل المرتفع دون سيطرة، وتعمل الدول ذات الدخل المنخفض والمتوسط دون أن يتم التخلي عنها، سيكون ضروريًا لتحقيق تكيف عادل وفعال مع تغير المناخ.

## 摘要

我们讨论了在气候变化适应和全球健康研究方面存在的一项伦理挑战。该项挑战源自涉及以下方面的两对直觉性认知：气候变化和健康研究方面的公平保障和责任分配。一对直觉性认知的持有者认为，考虑到高收入国家的历史排放量、不均衡资源分配和干预能力，它们应承担开展适应研究的责任。另一对直觉性认知的持有者则认为，考虑到中低收入国家的能动性、自决权、地方当局和合法性以及不成比例的气候和健康风险负担，它们也应承担开展此类研究的责任。这两对直觉性认知导致形成了相冲突的两种观点：一种从义务和援助的角度出发考虑问题，另一种则从能动性和权威的角度出发考虑问题。为了应对这种紧张局面，我们将责任划分为以下两种：(i) 促成适应研究的责任；和 (ii) 实施适应研究的责任。最终的责任分工结果既反映了各种公平保障方式,也彰显了辅助性原则的核心要素，即：不抛弃、不取代及合作与协调。因此，我们建议按照责任划分框架，将促成适应研究的责任（包括适应融资、能力建设和其他形式的支持）分配给高收入国家，并将实施适应研究的责任（包括在当地适应研究中设定优先顺序以及制定和实施其适应计划和政策）分配给中低收入国家。我们还在框架中建议划分出可由高收入国家、中低收入国家以及这两类国家内部的各级机构共同承担的第三种责任：共享适应责任。结论表明，在适应研究方面真正开展平等合作（即确保高收入国家促成而不主导适应研究，中低收入国家自主行动而不被抛弃）将是以公平有效的方式推动气候变化适应研究的重要保障。

## Резюме

Авторы обращаются к этической проблеме, возникающей в ходе исследований адаптации к изменениям климата и глобального здравоохранения. Проблема коренится в наличии двух наборов интуитивных представлений о справедливости и ответственности в контексте изменений климата и здоровья. Согласно одному из них, ответственность за исследования адаптации лежит на странах с высоким уровнем дохода с учетом их исторического уровня выбросов, непропорционального распределения ресурсов и способности осуществлять вмешательства. Другой возлагает ответственность на страны с низким и средним уровнем дохода, учитывая их субъектность, право на самоопределение, местные полномочия и легитимность, а также по соображениям диспропорционального бремени климатических рисков и рисков для здоровья. Такие интуитивные представления формируют противоречивые позиции: обязательства и помощь тянут в одном направлении, тогда как субъектность и полномочия – в другом. Чтобы разрешить это противоречие, авторы предлагают две формы ответственности: (і) ответственность за создание возможностей для адаптации и (ii) ответственность за реализацию адаптации. Достигаемое в результате разделение труда отражает разнообразие форм справедливости и соответствует основным элементам принципа субсидиарности, включая неоставление, непоглощение, а также сотрудничество и координацию. Таким образом, авторы предлагают рамочную концепцию, которая возлагает ответственность за создание возможностей для адаптации на страны с высоким уровнем дохода, включая финансирование адаптации, наращивание потенциала и другие формы поддержки, а ответственность за реализацию адаптации – на страны с низким и средним уровнем дохода, включая определение приоритетов в локальных исследованиях по адаптации, а также разработку и внедрение их адаптационных планов и политик. Эта рамочная концепция также предполагает и третью форму ответственности – совместную ответственность за адаптацию, которая возлагается совокупно на страны с высоким, низким и средним уровнями дохода и на субъектов различных уровней внутри этих стран. В заключение хотим отметить, что подлинное сотрудничество в области исследований адаптации, когда страны с высоким уровнем дохода не доминируют, а страны с низким и средним уровнями дохода действуют, не будучи оставленными без поддержки, станет очень важным условием справедливой и эффективной адаптации к изменениям климата.

## Introduction

The scale, severity and urgency of climate change require that humanity responds with complex actions at various scales.^1^ Although mitigating climate change by reducing greenhouse gas emissions receives the most attention, it is just one category of climate action in the policies that we must adopt.^2^ The stakes are high: health risks and impacts of climate change are substantial and increasing.^3^ The global scientific community must continue health advocacy for climate action,^4^ and expand research on the health impacts of climate change and strategies for coping with them. However, this research has significant ethical challenges. Here, we consider questions of justice and responsibility in health research as it relates to another important category of climate action: adaptation.

Mitigation limits or slows climate change by reducing greenhouse gas emissions, while adaptation helps us to live with climate change by reducing vulnerability and exposure to its hazards.^5^ Adaptation includes different changes at multiple levels, with no one-size-fits-all solutions,^6^ but can be defined as “the process of adjustment to actual or expected climate change and its effects to moderate or avoid harm or exploit beneficial opportunities.”^7^

Climate and health research is crucial for adaptation planning and policy. Although recent years have seen substantial progress in research on adaptation for health, knowledge gaps remain, including for adaptations to extreme heat, infectious disease, malnutrition and mental health.^5^ Notably, working group II of the Intergovernmental Panel on Climate Change states, “No universal standardized approach exists for monitoring or evaluating adaptation activities in the health sector.”^5^ This gap indicates research needs, including developing metrics, designing monitoring and evaluation frameworks, and implementing these frameworks in different systems.

There are also ethical gaps, which sometimes exacerbate challenges of scientific enquiry. Because the goals of adaptation are value-laden, different ethical (value-based) perspectives can lead to conflicting interpretations of an adaptation strategy’s adequacy and effectiveness, thereby complicating adaptation research and policy.^8^ Adaptation research, like adaptation itself, is ethically and politically fraught.^9^^,^^10^ Yet, the past 30 years of climate ethics scholarship has focused on mitigation and given less consideration to the ethical challenges of adaptation.^2^^,^^11^ Bioethics has begun to pay more attention to environmental issues, including climate change,^12^^,^^13^ but has addressed adaptation infrequently.^10^^,^^14^^–^^16^

Here, we advance the ethics of adaptation by considering questions of justice and responsibility in adaptation research, questions about what the adaptation research agenda should be, how it should be shaped, by whom and on what grounds. We examine ethical intuitions that pull in opposite policy directions, and we resolve this tension by proposing a practical framework that distinguishes two forms of responsibility relevant to adaptation research.

We discuss high-income countries and low- and middle-income countries. These classifications are imperfect as they obscure heterogeneity and oversimplify the range of relevant actors, but they help us think about responsibility systematically.

## Responsibility: opposed intuitions

Two pairs of ethical intuitions pull in opposite policy directions when assigning responsibility for adaptation research. While ethical intuitions are initial moral judgments, when examined and refined, they can reappear in a more formal guise as principles in academic literatures or embedded in frameworks and commitments that shape policy and practice. Table 1 maps each intuition onto these more precise expressions. One pair of intuitions emphasizes responsibilities of high-income countries; the other emphasizes responsibilities of low- and middle-income countries. We present these intuitions in simplified form as useful starting points. Exceptions exist, but these intuitions help bring key considerations into focus.

**Table 1:** Ethical intuitions and their real-world expressions

Ethical intuition	Corresponding ethics concepts and principles (in academic literature)	Corresponding formal expressions (in governance and policy documents)	Corresponding practical expressions (i.e. patterns of behaviour and practices that reflect the intuition)
Intuition 1: High-income countries are responsible for climate change adaptation	Burden-sharing justice (fair allocation of adaptation-related burdens): polluter pays principle, beneficiary pays principle, ability to pay principle Harm avoidance justice (preventing avoidable harms): high-income countries have duties to uphold rights and entitlements of individuals and communities in low- and middle-income countries to have and exercise the ability to adapt to climate change	United Nations Framework Convention on Climate Change (1992): common but differentiated responsibilities and respective capabilities Kyoto Protocol (1997–2005): common but differentiated responsibilities and respective capabilities Paris Agreement (2015): Article 2(2) (“common but differentiated responsibilities and respective capabilities, in light of national circumstances”), Article 7	Financial commitments by high-income countries to fund adaptation in low- and middle-income countries (e.g. Kyoto Adaptation Fund, 2001) Capacity-building (e.g. training, institutional strengthening) in adaptation research and planning Adaptation technology transfer (e.g. diffusion of know-how and equipment for irrigation systems, crop and soil management technologies and storm surge barriers)
Intuition 2: High-income countries are responsible for global health research	Compensatory justice Distributive justice Duties of beneficence and rescue Human rights and entitlement-based duties	UN Universal Declaration of Human Rights (1948): Article 25(1), Article 27 UN International Covenant on Economic, Social and Cultural Rights (1966): Article 2(1), Article 15(2) Research for Health Justice ethical framework Canadian Coalition for Global Health Research, principles for global health research: shared benefits, responsiveness	Funding calls and institutional strategies that explicitly prioritize disparity-reducing research questions Research portfolios sponsored by high-income countries targeting neglected high-burden conditions in low- and middle-income countries, with a public rationale tied to equity Reparative investments as state obligations Treaty-style advance commitments for products relevant to low- and middle-income countries Intellectual property and trade policy designed to reduce inequities in research and access to medicines, vaccines and diagnostics, among others Capacity-building programmes in low- and middle-income countries led and funded by high-income countries
Intuition 3: Low- and middle-income countries are responsible for climate change adaptation	Principle of self-determination Legitimacy and authority Thick conceptions of human flourishing (context-specific values) Recognitional justice Participatory justice Agents of justice (who realizes justice)	Paris Agreement (2015): Article 2(2), Article 7 United Nations Framework Convention on Climate Change Conference of the Parties (2011): Decision 5/CP.17 on National Adaptation Plans Green Climate Fund: no-objection procedure (funding aligns with a country’s climate priorities) Green Climate Fund: country ownership policy (nationally determined priorities) Adaptation Fund: direct access; national implementing entities	Legitimate authority over goal- and priority-setting in context Local determination of adaptation success criteria Local determination of acceptable risks, losses, and damages Local determination of implementation pathways
Intuition 4: Low- and middle-income countries are responsible for global health research	Principle of self-determination Legitimacy and authority Recognitional justice Participatory justice Agents of justice (who realizes justice) Authority to assign value (distinct from exploitation protections and benefit-sharing)	Council for International Organizations of Medical Sciences and World Health Organization (2016): guideline 7 Accountability for Research: legitimacy via fair process Research for Health Justice ethical framework H3Africa: framework and community engagement guidelines World Health Organization (2025): guidance on the ethics of health research priority setting	Participatory research models, community engagement and power sharing in research based in low- and middle-income countries Priority-setting processes in global health research led by or engaging local decision authority in low- and middle-income countries (not consultation only)

Note: Each ethical intuition is paired with (i) corresponding ethics concepts and principles (in academic literatures); (ii) their corresponding formal expressions in governance documents and policies; and (iii) their corresponding expressions in recognizable practices. Taken together, Intuitions 1 and 2 tend to assign special responsibility for climate change adaptation research to high-income countries and actors, while Intuitions 3 and 4 emphasize the authority and agency of low- and middle-income countries and actors. A tension arises when these pull in different directions, both of which are morally compelling.

### Intuition 1

Intuition 1 suggests that high-income countries are responsible for climate adaptation. These countries are responsible because of their historical emissions (polluter pays), their benefits from those emissions (beneficiary pays) and/or their greater capacity to prevent harms and secure benefits (ability to pay).^17^ Some views also appeal to human rights, grounding duties of the affluent in the claims of individuals and communities facing high vulnerability and exposure through no fault of their own.^18^^,^^19^ Each responsibility story relates to a different form of justice or moral obligation: (i) compensatory justice;^17^ (ii) distributive justice;^17^ (iii) a principle of rescue (a duty to help when the cost to the helper is acceptable and small relative to the benefit)^20^ or progressive consequentialism;^21^ and (iv) human rights.^18^^,^^19^ These arguments invoke two kinds of climate justice: one emphasizes the fair allocation of adaptation burdens among duty-bearers; the other emphasizes the goal of adaptation to avert avoidable harms.^22^ Despite theoretical differences, these viewpoints converge on the practical conclusion that high-income countries bear special responsibility for adaptation.

### Intuition 2

Intuition 2 suggests that high-income countries are responsible for global health research. Global health research is often justified by its role in reducing unjust health disparities, including by prioritizing the interests of communities in low- and middle-income countries.^23^ The responsibilities of high-income-countries can be based on: (i) compensatory justice, given historical and ongoing injustices;^24^ (ii) distributive justice, given the severity of disparities;^25^^–^^27^ (iii) duties of beneficence or rescue;^20^ or (iv) human rights obligations. Although these justifications are debated in ethics and political philosophy,^28^ they support the idea that high-income countries have justice-related responsibilities or obligations that shape the global health research agenda.

### Intuition 3

Intuition 3 suggests that low- and middle-income countries and their institutions are responsible for climate adaptation in the sense that they are the rightful agents of adaptation. Because adaptation is local in its aims and implementation, decisions about adaptation goals and means should be made by those bodies with legitimate authority within the local context, consistent with the right to self-determination. The plans and aims should reflect community- and identity-specific thick conceptions of human flourishing, that is, detailed, context-dependent, culturally embedded views shaped by particular values and traditions, rather than thin conceptions stated only in abstract or generic terms.^29^^,^^30^ This forward-looking sense and agency-based notion of responsibility recognizes agents living in low- and middle-income countries as the appropriate agents of response. Relatedly, recent work on agents of justice argues that adequate interpretations of justice must recognize the roles of less powerful actors in realizing justice, which “accords with the intuition that the project of justice takes all types” and “resonates with [their] self-understanding.”^31^ This view is also reflected in recent work stressing the need for greater representation of vulnerable communities in global climate change policy and practice.^32^

### Intuition 4

Intuition 4 suggests that low- and middle-income countries are responsible for global health research. Participatory justice demands that the people or communities directly affected by research decisions have a meaningful role in the decision-making process. This view means that low- and middle-income countries and communities in these countries are responsible for decisions about the value of research questions, their priority and the acceptable research risk. This justice requirement is distinct from fair selection of participants, protection from exploitation and benefit-sharing.^33^ Rather, this point concerns the authority to assign value and set priorities in research, grounded in participatory and recognitional justice. This view is reflected in long-standing calls for community engagement and recent proposals for power-sharing in research priority-setting.^34^^–^^37^

## Explaining the tension

The tension is between two plausible and intuitive perspectives on responsibility for adaptation research that pull in different policy directions. One emphasizes the responsibilities of high-income countries related to their historical emissions, benefits therefrom, and capacity to assist. The other emphasizes the agency and authority of low- and middle-income countries. Because adaptation is local and value-laden, low- and middle-income countries are the rightful decision-makers about the goals and priorities of adaptation research conducted in their contexts or intended for their benefit. Efforts led by high-income countries can be intrusive or illegitimate if unsolicited.

This situation generates a practical dilemma: either high-income countries take responsibility, in which case they may encroach upon local authority; or actors in low- and middle-income countries take responsibility without support, in which case the responsibilities of high-income countries go unfulfilled and the adaptation response may not be enough. To put the point another way, some intuitions pull us towards distributive and compensatory justice, while others pull towards recognitional and participatory justice. We cannot simply choose between these two perspectives of justice and responsibility because both are essential. The challenge is to combine them into a coherent framework that provides practical guidance for allocating responsibilities for health adaptation research and captures the distinct varieties of justice involved.

## Resolving the tension

To resolve the tension, we introduce a responsibility allocation framework that distinguishes two forms of responsibility relevant to adaptation research: adaptation-enabling responsibilities and adaptation-enacting responsibilities. The framework assigns roles to different agents in a way that reconciles the considerations identified earlier and connects each role to corresponding types of justice (compensatory, distributive, recognitional and participatory). The framework translates our ethical argument into practical guidance for allocating responsibilities for adaptation research. The resulting division of labour integrates distinct forms of justice and harmonizes rival conceptions of responsibility. Table 2 sets out distinct categories of responsibility along with the relevant agents and justice-based rationales, and illustrates how these apply in adaptation-related health research.

**Table 2:** Responsibility allocation framework for adaptation research

Category of responsibility	Definition	Primary agents	Varieties of justice	Subsidiarity elements	Supporting ethical intuitions	Supporting normative principles	Implementation examples
Adaptation-enabling	Responsibilities to perform actions or make policies that create (or are conducive to) new states of affairs in which successful climate adaptations become possible, more likely, less difficult, better in quality, or greater in quantity or scale	High-income countries	Distributive; compensatory	Non-abandonment	Intuition 1: High-income countries are responsible for climate change adaptation Intuition 2: High-income countries are responsible for global health research	Polluter pays; beneficiary pays; ability to pay; intervention-responsibility	Financing: dedicated funding for adaptation-related health research in climate-vulnerable settings (multi-year; predictable) Addressing global structural barriers to research and uptake (e.g. procurement, licensing and access, and administrative constraints on cross-border collaboration) Capacity-building and technical support: training, institutional support, research administration and ethics review capacity for climate–health research Data-sharing and research facilitation: access to climate services, satellite and remote sensing inputs, interoperable data sets, and analytic support Technology and method transfer: tools for surveillance, forecasting, early warning systems and evaluation Support for monitoring and evaluation infrastructure: metrics and indicators development, evaluation frameworks, and implementation research capacity
Adaptation-enacting	Responsibilities to perform actions or make policies that reduce the vulnerability or exposure to a climate hazard of a person, population, community, or some object of concern	Low- and middle-income countries	Recognitional; participatory	Non-absorption	Intuition 3: Low- and middle-income countries are responsible for climate change adaptation Intuition 4: Low- and middle-income countries are responsible for global health research	Self-determination; legitimacy; local authority; anti-paternalism	Locally led priority-setting for adaptation-related health research questions (what to study, for whom and why) Local definition of adaptation goals and effectiveness criteria (including trade-offs and locally valued outcomes) Selection of outcome measures and endpoints that reflect local values and context (not just donor-preferred metrics) Governance of acceptable risks, burdens and benefit arrangements in studies conducted in context Leadership in adaptation planning and implementation informed by research (how evidence is translated into programmes, policy and practice)
Shared and/or overlapping	Responsibilities borne jointly by agents based in high-income and low- and middle-income countries to cooperate and coordinate over time and across countries, institutions, sectors, and levels of governance so that adaptation and the research informing it can be more just and effective	High-income countries and low- and middle-income countries (jointly, for shared goals)	Distributive; compensatory; recognitional; and participatory	Cooperation and coordination	Intuitions 1–4	Solidarity and belonging; the common good; shared-goals requirement; closeness criterion; mutual accountability; stewardship; reciprocity; trust and trustworthiness	Core outcome sets; shared standards for outcomes and reporting templates for cross-site comparability Shared data standards; co-development and joint stewardship of data systems Shared monitoring and evaluation indicators; cross-site evaluation protocols (learning loops) International research collaborations with shared governance and accountability mechanisms Regional technical hubs and platforms to translate findings to new contexts Coordination to address national or local structural barriers to uptake of adaptation research and equitable employment of adaptation strategies Domestic cofinancing when feasible (e.g. low- and middle-income countries’ budgetary prioritizations aligned with their national adaptation plans)

Note: this analytical framework distinguishes adaptation-enabling responsibilities, adaptation-enacting responsibilities and shared adaptation responsibilities, and links each category to primary agents, relevant forms of justice, subsidiarity elements, supporting ethical intuitions and normative principles, and examples of their practical implementation in the context of climate change health-related adaptation research. Adapted in part from Zimm et al., 2024.^38^

Our enabling–enacting distinction aligns with previous scholarship distinguishing between first- and second-order responsibilities.^22^ We also use insights from another justice framework for climate research and policy discussions.^38^ We adapt and simplify this other framework for purposes of adaptation research, omitting some categories (e.g. transitional justice) and units of analysis (patterns, metrics and scope of justice), while adding responsibility-specific elements.

### Enabling and enacting

 Enabling adaptation includes actions or policies that create (or are conducive to) a new situation in which successful climate adaptation becomes possible, more likely, less difficult, better in quality, or greater in quantity or scale. Enabling adaptation includes financing, capacity-building, data-sharing, research facilitation, technology transfer, and training that informs and improves a community’s adaptation planning and decision-making. Enabling adaptation can include more systematic interventions. For example, the Dutch Delta Program, a planning approach adopted in Viet Nam (Mekong Delta Plan), Bangladesh (BDP2100) and other low- and middle-income countries, includes a special envoy to share knowledge and advise on legal and financial frameworks.^39^ Enabling adaptation does not directly reduce vulnerability or exposure to health hazards related to climate change. Instead, this approach makes it possible (or easier) for the enabled to adapt.

Enacting adaptation includes actions or policies that reduce vulnerability or exposure to a climate hazard for persons, communities or some object of their concern. Here, we are setting aside maladaptation, where situations worsen despite the best of intentions.^40^ Enacting adaptation is what directly reduces vulnerability, exposure or resilience; enabling adaptation creates the conditions under which those changes can be achieved.

Our distinction parallels first-order and second-order responsibilities proposed earlier, including the second-order responsibility of enablement.^22^ In this scenario, agents might be unwilling to comply with their first-order responsibility to reduce greenhouse gas emissions unless enabling conditions are put in place (e.g. affordable low-carbon alternatives). In the mitigation examples given, one can affect compliance by: (i) facilitating research into clean technologies, new energy sources or increasing energy efficiency; and (ii) disseminating those innovations widely to make reducing emissions easier.^22^ The point applies to adaptation as well. The first-order responsibilities of low- and middle-income countries to adapt can be shaped by what other agents do. High-income countries can enable adaptation by: (i) facilitating research into climate–health risks in low- and middle-income countries and strategies for reducing vulnerability and exposure to those hazards; and (ii) transferring resources (funds, data technology, methods and training) to empower adaptation planning and implementation.

Importantly, as noted elsewhere, enablement not only helps agents fulfil preexisting first-order responsibilities, it can also create first-order responsibilities.^22^ For example, transferring clean technology can enable development, thereby creating an opportunity and responsibility to develop without making climate change worse.^22^ In the context of adaptation, this condition means that when a high-income country fulfils its enabling responsibilities in ways that increase the adaptive capacity and opportunities of a low- and middle-income country, the enabled country may thereby acquire new adaptation-enacting responsibilities. As the adaptation capacities and opportunities of agents increase, these agents can be assigned new adaptation-enacting responsibilities, that is responsibilities they did not have before being enabled.

Finally, although our focus is on the responsibilities of high-income countries and low- and middle-income countries, high-income countries are not the only ones to have adaptation-enabling responsibilities. For example, individuals and institutions in low- and middle-income countries can enable adaptation within their own countries and communities; research in low- and middle-income countries can also enable high-income countries to adapt; and high-income countries, and agents within them, have both self-directed enabling and enacting responsibilities. These responsibilities exist within nations as well as between them, and can be assigned to a wide variety of agents, not just sovereign states. Our framework emphasizes high-income countries’ adaptation-enabling responsibilities because they have overlapping justice-based reasons to provide enabling support and because the framework is intended to identify their responsibilities in global adaptation. Equally, our framework emphasizes low- and middle-income countries’ adaptation-enacting responsibilities, although high-income countries must adapt as well.

### Why these are responsibilities

To call something a responsibility and to ascribe it to an agent is to say that the agent ought to do it, has the ability or opportunity to do it, and is accountable or should be responsive to others’ appraisals of whether and how it is done. But why do we think high-income countries and low- and middle-income countries have these responsibilities?

We can divide the problem of adaptation responsibility into two questions: (i) what actions should be taken to prevent or reduce harms related to climate change? and (ii) who should carry the costs of taking those actions?^41^ This division parallels our own questions: (iii) who is responsible for enacting adaptation? and (iv) who is responsible for enabling adaptation?

Regarding enablement responsibilities, question (iv) above, we take a pragmatic approach. Multiple theories already proposed converge on assigning to high-income countries special responsibilities to enable adaptation in the low- and middle-income countries. Consider adaptation financing. One view is to treat adaptation finance as a matter of compensatory justice, based on harmful historical emissions that need compensation.^17^^,^^41^ A second view grounds the obligation to finance adaptation in distributive justice, emphasizing assistance from the fortunate to the less fortunate independent of causal responsibility for climate change.^42^ A third view is based on the current ability to prevent avoidable harms through intervention-responsibility.^43^ Intervention-responsibility is defined as: “Agent A is IR [intervention-responsible] for state of affairs S when 1) S is undesirable, 2) A could significantly mitigate S without excessive cost.”^43^ This principle ascribes responsibilities to high-income countries to finance adaptation in low- and middle-income countries.

Despite their differences, all three views converge on ascribing the responsibilities of adaptation finance to affluent individuals, affluent collectives and high-income countries. The affluent should urgently meet the obligations these theories convergently entail, setting aside deeper theoretical disagreement for later. This pragmatic attitude is not a claim that the philosophical disputes are unimportant, but rather a claim about the urgent and severe need for adaptation and the adequacy of the argument from convergence. Financing, like adaptation research, enables adaptation in the world’s most climate-vulnerable communities. These adaptation-enabling responsibilities should be borne by high-income countries and ought to be fulfilled now, even if we disagree about why.

### Subsidiarity and adaptation

Regarding the question of adaptation-enacting responsibilities, question (iii) above, we find a compelling answer in the principle of subsidiarity, according to which, “decision-making authority primarily lies with the level of governance closest to the issue or the community’s needs.”^44^ Subsidiarity advocates a distribution of power that aligns with our framework’s division of labour: low- and middle-income countries, and agents based in within them, have authority to decide local climate adaptation agendas by virtue of their right to self-determination and unique claim to legitimacy, and because adaptation aims at particular, community-specific projects and goals that must be articulated in terms of the adapting community’s conception of human flourishing.^29^^,^^30^

Although a full exploration of subsidiarity is beyond our scope, it is useful to consider its core elements. Based on previous research, subsidiarity comprises: (i) non-abandonment; (ii) non-absorption (an agency-protecting constraint); and (iii) cooperation and coordination among those agents participating in a process and sharing a common goal.^45^ As a structural principle of justice, higher levels of governance should provide assistance that responds to local needs and values, often framed as assistance on explicit request, rather than allowing donor agendas or market forces to dictate.^46^

This structure is precisely what is needed to resolve the practical dilemma we identified earlier. Subsidiarity shows how to avoid both deserting the vulnerable and displacing their agency. Non-abandonment connects with the responsibilities of high-income countries to enable adaptation in low- and middle-income countries through financing, capacity-building and other forms of support. At the same time, non-absorption constrains that enablement so that it does not displace the authoritative agency of low- and middle-income countries over adaptation goals, research priorities, and implementation. In short, high-income countries must enable adaptation without dominating and low- and middle-income countries must enact adaptation without being deserted.

The element of cooperation and coordination matters because adaptation-related health research is not a set of one-way, bilateral transactions. Adaptation research often requires sustained cooperation and coordination between countries, across institutions, sectors and levels of governance, and over time. This feature of adaptation research suggests a third category of adaptation responsibility, which is shared responsibilities (Table 2). Adaptation research means sharing responsibilities: shared standards for outcome sets and reporting templates to allow for cross-site comparisons of adaptation strategies; shared data systems; shared accountability mechanisms; and international hubs to translate findings to new contexts. The closeness criterion of subsidiarity helps assign these responsibilities by identifying and prioritizing coordinators who are geographically or jurisdictionally near the adapting community, or closely related to the problem through relevant experience, expertise and capacity.^45^ Proximity and expertise may not always coincide, but coordination and cooperation always require sharing common goals with the adapting community and a commitment to their achievement.

Subsidiarity is a core structural feature of our framework for justice and responsibility in health adaptation research. High-income countries have special responsibilities to enable adaptation (non-abandonment); low- and middle-income countries have special responsibilities to enact adaptation (non-absorption); and both have shared responsibilities to make adaptation research, policy and practice more just and effective (cooperation and coordination).^45^^,^^46^

## Clarifications

We have proposed a division of responsibilities for adaptation research. Because ours is a general framework rather than a step-by-step policy manual, it invites two concerns: that it is too abstract to guide practice, and that it relies on concepts that oversimplify a complex world. We address both concerns here.

First, as noted, in referring to high-income countries and low- and middle-income countries we are using imperfect categories. These classifications are widely used in global health policy and research, but they can obscure ethically relevant differences within and between countries,^47^ and can imply a view that sovereign states are the only relevant agents. Similar concerns arise in climate ethics and policy: high-income countries vary in historical and present-day emissions, and some low- and middle-income countries contribute substantially to current emissions or have substantial adaptive capacity, while climate vulnerability is uneven across both groups.^48^^,^^49^ Our argument, therefore, should not be read as a claim that all high-income countries (or all low- and middle-income countries) have identical responsibilities. Rather, our framework identifies types of responsibility, which are enabling, enacting and shared, and asks which agents in a given context are best positioned to bear them and on what basis. In some contexts, certain low- and middle-income countries (or agents within them) have important enabling responsibilities and attribution of responsibility shifts over time as capacities change.

Second, our framework does not say how best to design or implement adaptation in every context. The framework clarifies what justice requires when we are pulled in different directions by plausible and intuitive moral judgments. Subsidiarity helps reconcile these demands: the principle requires reliable support where stakes are high, the protection of agency from domination, and cooperation and coordination to ensure that research can inform adaptation. This principle has a practical implication for research governance: funding, capacity-building and partnership design should be organized so that enablement is responsive to locally voiced goals, while authority for priority-setting and decision-making on adaptation research goals remain with legitimate local agents.

Third, important complexities remain, including the internal dynamics within low- and middle-income countries, or any country for that matter. Political agency may be fragmented, contested, or captured; conflicts of interest, power imbalances, exclusion, or weak institutional legitimacy can undermine justice at local or national levels.^50^ Against this backdrop, hard questions arise. Whose values and priorities are treated as authoritative? How is adaptation enacted in practice? What implementation challenges must be overcome? Our framework does not assume simple realities. Instead, the framework clarifies what is at stake in these challenges. Non-absorption forbids replacing local authority with donor authority, but it does not require treating any particular domestic actor as authoritative irrespective of legitimacy.

Finally, we emphasize that the enabling–enacting distinction permits overlap. In practice, adaptation research involves shared responsibilities and dynamic collaborations. Enablement can strengthen local capacity, local enactment can create knowledge that reshapes the situation, and both sides can be jointly responsible for coordination. Our framework’s value is not that it eliminates complexity, but that it prevents two common failures in global health research, which are domination and abandonment, while providing a method for assigning responsibilities in a world where adaptation has become more urgent, yet agency still matters.

## Conclusion

Financing adaptation research is not the only responsibility high-income countries have to enable adaptation in low- and middle-income countries, but it is an important one. Many other actions are required to enable vulnerable communities to adapt to climate change. Future work, both empirical (descriptive) and normative (ethical), is needed to discover which adaptation-enabling responsibilities to prioritize and for what reasons. Meanwhile, priority-setting in adaptation research must be done in such a way that high-income countries enable low- and middle-income countries to adapt without deciding or doing that adaptation for them. Achieving this ideal will require genuine forms of collaboration, solidarity and adapting together.

We encourage future work from other scholars to build this diversity and complexity into our more general approach. We believe that our framework is complementary to more detailed descriptions of particular settings, and revisable in light of such details. The main contribution of the framework is that it helps researchers, research ethicists and policy-makers think about how their work in climate and health is connected to justice, how to embed commitments to justice more deeply in their work and to consider what forms of justice ground the responsibilities they are taking.

## References

[1] Summary for policymakers. In: Lee H, Romero J, editors. Climate change 2023: synthesis report. Geneva: Intergovernmental Panel on Climate Change; 2023. pp. 1–34. 10.59327/IPCC/AR6-9789291691647

[2] Jamieson D. Adaptation, mitigation, and justice. In: Sinnott-Armstrong W, Howarth RB, editors. Perspectives on climate change: science, economics, politics, ethics. Amsterdam: Elsevier; 1996. pp. 217–48.

[3] Romanello M, Walawender M, Hsu SC, Moskeland A, Palmeiro-Silva Y, Scamman D, et al. The 2025 report of the Lancet Countdown on health and climate change: climate change action offers a lifeline. Lancet. 2025 Dec 13;406(10521):2804–57. 10.1016/S0140-6736(25)01919-1 41175887

[4] Allan JI. Advocating for health and climate. Bull World Health Organ. 2023 Feb 1;101(2):158–60. 10.2471/BLT.22.289283 36733631 PMC9874372

[5] Cissé G, McLeman R, Adams H, Aldunce P, Bowen K, Campbell-Lendrum D, et al. Health, wellbeing, and the changing structure of communities. In: Pörtner H-O, Roberts DC, Tignor M, Poloczanska ES, Mintenbeck K, Alegría A, et al., editors. Climate change 2022: impacts, adaptation, and vulnerability. Contribution of Working Group II to the Sixth Assessment Report of the Intergovernmental Panel on Climate Change. Cambridge: Cambridge University Press; 2023. pp. 1041–170., 10.1017/9781009325844.009

[6] Introduction: adaptation and resilience – loss and damage [internet]. Bonn: United Nations Framework Convention on Climate Change. Available from: https://unfccc.int/topics/adaptation-and-resilience/the-big-picture/introduction [cited 2026 Jan 11].

[7] Dale L. Climate change adaptation: an Earth Institute sustainability primer. New York: Columbia University Press; 2022. 10.7312/dale19916

[8] Singh C, Iyer S, New MG, Few R, Kuchimanchi B, Segnon AC, et al. Interrogating “effectiveness” in climate change adaptation: 11 guiding principles for adaptation research and practice. Clim Dev. 2022;14(7):650–64. 10.1080/17565529.2021.1964937

[9] Sheather J, Littler K, Singh JA, Wright K. Ethics, climate change and health – a landscape review. Wellcome Open Res. 2023 Aug 14;8:343. 10.12688/wellcomeopenres.19490.1 37692130 PMC10483174

[10] Ferguson K. Climate change, health, and the ethics of adaptation. In: Richie C, editor. The Oxford handbook of environmental bioethics. New York: Oxford University Press. Forthcoming 2026.

[11] Jamieson D. Reason in a dark time: why the struggle against climate change failed—and what it means for our future. New York: Oxford University Press; 2014. 10.1093/acprof:oso/9780199337668.001.0001

[12] Resnik DB. Bioethics and global climate change. Bioethics Forum. 2009;39(3):1. 19484138 PMC2688386

[13] Macpherson CC. Climate change is a bioethics problem. Bioethics. 2013 Jul;27(6):305–8. 10.1111/bioe.12029 23718702

[14] Ferguson K. The health reframing of climate change and the poverty of narrow bioethics. J Law Med Ethics. 2020 Dec;48(4):705–17. 10.1177/1073110520979381 33404344

[15] Anderson W, Troy J, Lucas T, Capon A, Komesaroff P, Callicott JB, et al. Bioethics for the planet. Lancet. 2025 Aug 23;406(10505):881–4. 10.1016/S0140-6736(25)01068-2 40749696

[16] Arguedas Ramírez G, Bruce L, Dabbagh H, Earp BD, Shamsi Gooshki E, Ma Y, et al. Global bioethics bulletin: dispatches on climate justice. JME Practical Ethics. 2026;2(1):e000083. 10.1136/jmepb-2025-000083

[17] Caney S. Climate justice. In: Zalta EN, editor. Stanford encyclopedia of philosophy [internet]. Stanford: Stanford University; 2021. Available from: https://plato.stanford.edu/archives/win2021/entries/justice-climate/ [cited 2025 Jun 1].

[18] Caney S. Cosmopolitan justice, responsibility, and global climate change. Leiden J Int Law. 2005;18(4):747–75. 10.1017/S0922156505002992

[19] Byskov MF. The right to climate adaptation. Ethical Theory Moral Pract. 2024;27(4):477–504. 10.1007/s10677-024-10438-z

[20] Singer P. Famine, affluence and morality. Philos Public Aff. 1972;1(3):229–43. 10.1007/s10677-024-10438-z

[21] Jamieson D, Elliot R. Progressive consequentialism. Philos Perspect. 2009;23(1):241–51. 10.1111/j.1520-8583.2009.00169.x

[22] Caney S. Two kinds of climate justice: avoiding harm and sharing burdens. J Polit Philos. 2014;22(2):125–49. 10.1111/jopp.12030

[23] Benatar SR, Singer PA. Responsibilities in international research: a new look revisited. J Med Ethics. 2010;36(4):194–7. PMID:20338927 10.1136/jme.2009.032672 20338927

[24] Pogge TW. Responsibilities for poverty-related ill health. Ethics Int Aff. 2002;16(2):71–9. 10.1111/j.1747-7093.2002.tb00398.x 15709280

[25] Daniels N. Justice, health, and healthcare. Am J Bioeth. 2001 Spring;1(2):2–16. 10.1162/152651601300168834 11951872

[26] Powers M, Faden R. Social justice: the moral foundations of public health and health policy. Oxford: Oxford University Press; 2006.

[27] Sharp D, Millum J. Prioritarianism for global health investments: identifying the worst off. J Appl Philos. 2015;35(1):112–32. 10.1111/japp.12142

[28] Hunter D, Dawson AJ. Is there a need for global health ethics? For and against. In: Benatar S, Brock G, editors. Global health and global health ethics. Cambridge: Cambridge University Press; 2011. pp. 77–88. 10.1017/CBO9780511984792.007

[29] Hourdequin M. Ethics, adaptation, and the Anthropocene. Ethics Policy Environ. 2021;24(1):60–74. 10.1080/21550085.2021.1904530

[30] Heyward C. Ethics and climate adaptation. In: Gardiner SM, Thompson A, editors. The Oxford handbook of environmental ethics. New York: Oxford University Press; 2017. pp. 474–86.

[31] Hickey C, Meijers T, Robeyns I, Timmer D. The agents of justice. Philos Compass. 2021;16(10):e12770. 10.1111/phc3.12770

[32] Byskov MF, Hyams K. Introduction: representing vulnerable communities and future generations in the face of climate change. Ethics Int Aff. 2022;36(2):135–6. 10.1017/S0892679422000211

[33] Luna F, Macklin R. Research involving human beings. In: Kuhse H, Singer P, editors. A companion to bioethics. 2nd ed. Malden, MA: Wiley-Blackwell; 2009. 10.1002/9781444307818.ch38

[34] Levine RJ. Ethical issues in international vaccine research and development. Yale J Biol Med. 2005 Oct;78(5):231–7. PMCID:2259157 17132330 PMC2259157

[35] Emanuel EJ, Wendler D, Killen J, Grady C. What makes clinical research in developing countries ethical? The benchmarks of ethical research. J Infect Dis. 2004 Mar 1;189(5):930–7. 10.1086/381709 14976611

[36] Pratt B. Sharing power in global health research: an ethical toolkit for designing priority-setting processes that meaningfully include communities. Int J Equity Health. 2021 May 25;20(1):127. 10.1186/s12939-021-01453-y 34034747 PMC8145852

[37] Pratt B. Research for Health Justice: an ethical framework linking global health research to health equity. BMJ Glob Health. 2021 Feb;6(2):e002921. 10.1136/bmjgh-2020-002921 33589418 PMC7887339

[38] Zimm C, Mintz-Woo K, Brutschin E, Hanger-Kopp S, Hoffmann R, Kikstra JS, et al. Justice considerations in climate research. Nat Clim Chang. 2024;14(1):22–30. 10.1038/s41558-023-01869-0

[39] Oliver L. Countries around the word are looking to the Netherlands to help them deal with flooding and water crisis. Here’s why [internet]. Rotterdam and Nairobi: Global Center for Adaptation; 2019. Available from: https://gca.org/countries-around-the-world-are-looking-to-the-netherlands-to-help-them-deal-with-flooding-and-water-crisis-heres-why/ [cited 2026 Jan 5].

[40] Schipper ELF. Maladaptation: when adaptation to climate change goes very wrong. One Earth. 2020;3(4):409–14. 10.1016/j.oneear.2020.09.014

[41] Baer P. Adaptation: who pays whom? In: Adger WN, Paavola J, Huq S, Mace MJ, editors. Fairness in adaptation to climate change. Cambridge: MIT Press; 2006. pp. 131–54. 10.7551/mitpress/2957.003.0012

[42] Meyer LH, Roser D. Climate justice and historical emissions. Crit Rev Int Soc Polit Philos. 2010;13(1):229–53. 10.1080/13698230903326349

[43] Jamieson D. Responsibility and climate change. Glob Justice Theory Pract Rhetor. 2015;8(2):23–42. 10.21248/gjn.8.2.86

[44] Atuire CA. Presidential address. International Association of Bioethics 17th World Congress of Bioethics. Religion, culture and global bioethics, 3–6 June 2024, Doha, Qatar [internet video]. San Bruno: YouTube; 2024. Available from: https://www.youtube.com/live/gMeIMR8AjK8 [cited 2026 Jan 11].

[45] de Campos-Rudinsky TC, Canales M. Global health governance and the principle of subsidiarity: in defense of a robust decentralization approach. Int J Const Law. 2022;20(1):177–203. 10.1093/icon/moac002

[46] de Campos-Rudinsky TC, Bosha SL, Wainstock D, Sekalala S, Venkatapuram S, Atuire CA. Decolonising global health: why the new Pandemic Agreement should have included the principle of subsidiarity. Lancet Glob Health. 2024 Jul;12(7):e1200–3. 10.1016/S2214-109X(24)00186-4 38735301 PMC7618881

[47] Lencucha R, Neupane S. The use, misuse and overuse of the “low-income and middle-income countries” category. BMJ Glob Health. 2022 Jun;7(6):e009067. 10.1136/bmjgh-2022-009067 35672116 PMC9185671

[48] Vulnerability. Country rankings [internet]. In: Notre Dame Global Adaptation Initiative (ND-GAIN); 2023. Available from: https://gain-new.crc.nd.edu/ranking/vulnerability [cited 2025 Dec 31].

[49] Ritchie H, Rosado P, Roser M. Per capita, national, historical: how do countries compare on CO_2_ metrics? [internet]. Our World in Data; 2023. Available from: https://ourworldindata.org/co2-emissions-metrics [cited 2025 Dec 28].

[50] Resnik DB. Environmental justice and climate change policies. Bioethics. 2022 Sep;36(7):735–41. 10.1111/bioe.13042 35488802 PMC9391311

